# The Practical Medical Series of Year Books

**Published:** 1903-02

**Authors:** 


The Practical Medical Series of Year Books. Comprising ten volumes on the year's Progress in Medicine and Surgery. Issued monthly under the general editorial charge of Gustavus P. Head, M. D., Professor of Laryngology and Rhinology in the Chicago Post-Graduate Medical School. Volume VIII. Pediatrics and Orthopedic Surgery. Edited by W. S. Christopher, M. D., John Ridlon, A. M., M. D., Samuel J. Walker, A. B., M. D., 1902. Chicago: The Year Book Publishers. [Price, $1.25; entire series, $7.50.]

If all the books of this series are as excellent as this one the Year Book Publishers are to be congratulated in having prepared so desirable a work. The literature of pediatrics for the year is here classified under appropriate headings and abstracts made of the leading articles. The volume is of convenient size for handling and the collection of all the literature pertaining to a subject in one volume gives the system a decided advantage over the cumbrous one-volume year book. M. J. F.

